# Source-Free Active Domain Adaptation for Brain Tumor Segmentation via Mamba and Region-Level Uncertainty

**DOI:** 10.3390/brainsci16030300

**Published:** 2026-03-08

**Authors:** Haowen Zheng, Che Wang, Yudan Zhou, Congbo Cai, Zhong Chen

**Affiliations:** 1 Department of Electronic Science, Xiamen University, Xiamen 361005, China; hwzheng01@stu.xmu.edu.cn (H.Z.); 33320230156265@stu.xmu.edu.cn (C.W.); cbcai@xmu.edu.cn (C.C.); 2 Institute of Artificial Intelligence, Xiamen University, Xiamen 361005, China; ydzhou@stu.xmu.edu.cn

**Keywords:** brain tumor segmentation, domain adaptation, active learning, Mamba, uncertainty estimation

## Abstract

**Background/Objectives:** Accurate brain tumor segmentation from MRI is crucial for diagnosis but faces challenges like domain shifts across medical centers, data privacy constraints, and high annotation costs. While source-free active domain adaptation (SFADA) emerges as a promising solution to these issues, existing approaches often overlook the inherent structural complexity in tumor regions. **Methods:** We propose a novel SFADA framework composed of two major contributions. First, we introduce a Region-level Uncertainty-Guided Sample Selection (RUGS) strategy, enabling the identification of the most informative target-domain samples in a single inference pass. Second, we present the Source-Free Active Domain Adaptation Network (SFADA-Net), a Mamba-driven segmentation model equipped with a dual-path multi-kernel convolution module for enhanced local feature interaction and a structure-aware prompted Mamba module for capturing global spatial relationships. **Results:** Extensive evaluations across one source domain dataset (BraTS-2021) and three target domain datasets (BraTS-SSA, BraTS-PED, and BraTS-MEN 2023) demonstrate the superior adaptability of the proposed method, achieving consistently high segmentation accuracy across domains. With only 5% annotation budget, our framework consistently outperforms state-of-the-art segmentation and domain adaptation methods, achieving robust segmentation accuracy across diverse domains and approaching the performance of fully supervised learning. **Conclusions:** The proposed method achieves superior accuracy in brain tumor region segmentation and precise boundary delineation under a limited annotation budget. It effectively mitigates domain shift while fully complying with data privacy regulations. Consequently, our framework relieves manual annotation bottlenecks and accelerates the cross-center deployment of accurate diagnostic tools, facilitating the clinical application of domain adaptation.

## 1. Introduction

Gliomas are one of the most common primary brain tumors and pose a serious threat to human health [1]. Magnetic resonance imaging (MRI), with its advantages of non-invasiveness, high-quality imaging, and absence of skull artifacts, has become the central tool in the diagnosis of brain tumors [2,3,4,5]. However, in clinical practice, radiologists mainly delineate tumor regions manually on MRI, which is time-consuming, labor-intensive, and prone to error and inter-observer variability, particularly in resource-limited settings [6]. These limitations may compromise delineation accuracy and even lead to treatment failure, underscoring the urgent need for efficient and robust automated solutions [7].

While numerous automated approaches, particularly convolutional neural networks (CNNs) and Transformers, have achieved remarkable progress in brain tumor segmentation, they are limited by insufficient global representation and high computational costs, respectively [8,9,10]. In this context, the emerging Mamba architecture has attracted wide attention, showing strong performance in image segmentation tasks. VM-UNet [11] first integrated Vision Mamba into the U-Net [12] architecture, while Swin-UMamba [13] combined Mamba with the Swin Transformer. Both approaches improve segmentation accuracy. However, brain tumor MRI images often have complex textures, irregular shapes, and low contrast with surrounding tissues. These characteristics make feature segmentation and boundary delineation challenging, placing higher demands on the robustness and fine feature perception ability of Mamba in real-world applications [14,15].

Although deep learning has achieved remarkable progress in brain tumor segmentation, its clinical application is still limited by several factors, including the high cost of obtaining annotated data, significant cross-center domain shifts, data scarcity in low-resource regions, and strict constraints on data security privacy [16,17,18]. To address these challenges, a variety of domain adaptation strategies have been proposed. Unsupervised domain adaptation (UDA) [19] uses labeled source data but remains source-dependent. Source-free domain adaptation (SFDA) [20] eliminates source data access during training but faces performance gaps compared to supervised models [21,22]. Semi-supervised domain adaptation (SSDA) enhances generalization by labeling limited target samples, yet its random selection ignores variations in sample information and difficulty [23,24]. Source-free active domain adaptation (SFADA) extends the SFDA framework by incorporating active learning. It actively selects a small set of target-domain samples for manual annotation and then fine-tunes the model in a semi-supervised way, without accessing source-domain data [25]. This strategy reduces annotation costs while maintaining data privacy.

Despite its potential, current methods present notable gaps in effectively bridging source and target domains. Current SFADA methods often select target samples based on model uncertainty or feature distribution [25,26,27]. They do not adequately reflect the differences in structural complexity and discriminative difficulty among brain tumor regions. In addition, these methods tend to depend strongly on the performance of the initial segmentation model, which limits further improvements in domain adaptation effectiveness.

To address the above issues, we propose a novel uncertainty-based SFADA framework designed to handle the structural complexity of brain tumors. Specifically, we propose a Region-level Uncertainty-Guided Sample Selection (RUGS) strategy. It includes Region-level Uncertainty Aggregation (RUA) and Uncertainty-Guided Core-set Selection (UGCS). The former aggregates high-uncertainty regions in slices. The latter ensures sample diversity while prioritizing samples that the model finds most uncertain. RUGS requires no source domain data and completes sample selection in one inference pass. To further address the limitations of backbones in segmentation performance, we introduce Source-Free Active Domain Adaptation Network (SFADA-Net), a Mamba-based backbone model. The core components of SFADA-Net are the Dual-Path Multi-Kernel Convolution (DPMKC) module and the Structure-Aware Prompted Mamba (SAPM) module. DPMKC uses dual-path cascaded convolutions to enhance local feature extraction. SAPM applies grouped structure-aware processing and adds prompts to improve Mamba’s ability to capture spatial structures and handle anti-causal modeling. Overall, the combination of RUGS and SFADA-Net effectively narrows the performance gap between source-free adaptation and supervised learning under very low annotation budgets.

The main contributions of this work are as follows:

- To our knowledge, this is the first work to apply SFADA to cross-center brain tumor segmentation. It explores a new solution and improves domain adaptation accuracy.
- We introduce the RUGS strategy, which identifies the most informative target-domain samples for annotation without requiring any source data, thereby significantly reducing labeling cost.
- We propose SFADA-Net, a Mamba-based segmentation model, and train it in a semi-supervised setting using samples selected by RUGS, which enhances domain adaptation performance.
- We conducted extensive experiments on three target-domain datasets and demonstrated that our framework achieved more accurate segmentation and better generalization than related state-of-the-art domain adaptation methods.

## 2. Methods

This section presents the proposed framework for SFADA. Section 2.1 formulates the problem setting, and Section 2.2 introduces the RUGS strategy. Subsequently, Section 2.3 and Section 2.4 detail the SFADA-Net architecture and the semi-supervised training objectives.

### 2.1. Problem Setting and Notation

We consider SFADA setting for brain tumor segmentation. Let $D^{s}={\left\{x_{n}^{s},\;y_{n}^{s}\right\}}_{n=1}^{N_{s}}$ denote the source-domain dataset consisting of $N_{s}$ labeled samples, where $x_{n}^{s}$ is the image and $y_{n}^{s}$ is the corresponding pixel-wise label from the label space $Y=\left\{0,\;1,\ldots C-1\right\}$. In the adaptation phase, we are given a target domain dataset $D^{t}={\left\{x_{n}^{t}\right\}}_{n=1}^{N_{t}}$ with $N_{t}$ unlabeled samples. We assume a domain shift exists between the source and target distributions, i.e., $P^{s}\left(x,y\right)\neq P^{t}\left(x,y\right)$. Crucially, the source dataset $D^{s}$ is inaccessible due to privacy concerns; only the source-pretrained model and the unlabeled target data $D^{t}$ are available.

We aim to select a small yet informative subset of target samples, denoted as $D_{l}^{t}\subseteq D^{t}$, for manually annotated under an annotation budget B. The remaining samples constitute the unlabeled set $D_{u}^{t}=D^{t}\backslash D_{l}^{t}$. Using the labeled set $D_{l}^{t}$ and the unlabeled set $D_{u}^{t}$, we adapt the pretrained model to optimize segmentation performance on the target domain.

### 2.2. Region-Level Uncertainty-Guided Sample Selection Strategy

**Figure 1:**
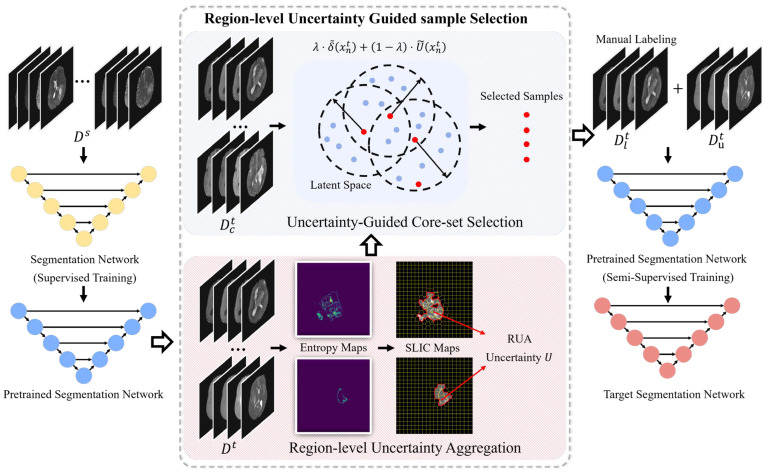
Workflow of the proposed RUGS strategy. Starting with the source-pretrained model, the framework applies RUA to estimate uncertainty U and form a candidate set $D_{c}^{t}$. Subsequently, UGCS identifies the most valuable samples for annotation. Finally, we leverage both the labeled set $D_{l}^{t}$ and the unlabeled set $D_{u}^{t}$ to adapt the model using semi-supervised learning.

To select the most valuable samples for annotation, we propose the RUGS strategy, which consists of two sequential components: RUA and UGCS, as illustrated in Figure 1. RUA first filters the model predictions to identify target-domain samples with high regional uncertainty. From this pool, UGCS selects a refined subset that balances uncertainty and diversity. Once annotated by experts, we incorporate these chosen samples into SFADA-Net for semi-supervised training.

#### 2.2.1. Region-Level Uncertainty Aggregation

Given an unlabeled input image $x_{n}^{t}\in D_{u}^{t}$, the pretrained segmentation network $f^{s}\left(\cdot ;\theta \right)$ outputs the logits for each pixel. For a specific pixel i in the image spatial domain Ω, the entropy $E_{i}$ at that pixel is computed based on the logits via the softmax function as follows [28]:

(1) $$E_{i}\left(x_{n}^{t}\right)=-\sum_{c=1}^{C}\underset{c}{\mathrm{softmax}}\left(f_{i}^{s}\left(x_{n}^{t}\right)\right)\log\left(\underset{c}{\mathrm{softmax}}\left(f_{i}^{s}\left(x_{n}^{t}\right)\right)\right),$$

where $\underset{c}{softmax}$ denotes the softmax output for class c, $f_{i,c}^{s}\left(x_{n}^{t}\right)$ denotes the logit for class c at pixel i.

To aggregate pixel-wise uncertainty into region-level representations, we propose RUA method. This method includes the following steps. First, we apply the Simple Linear Iterative Clustering (SLIC) algorithm [29] to partition the entropy map E into $N_{s}$ superpixel regions ${\left\{R_{j}\right\}}_{j=1}^{N_{s}}$, satisfying $\overset{N_{s}}{\underset{j=1}{⋃}}R_{j}=\Omega$ and $R_{j}\cap R_{j^{\prime }}=\emptyset$ for $j\neq j^{\prime }$. Compared with other superpixel methods, we choose SLIC because of its fast computation speed, good boundary adherence, and controllable number of segmented regions. This is particularly important for capturing complex tumor boundaries in medical images. We set the number of superpixel regions $N_{s}$ to 400 and the compactness parameter to 0.05, which achieves finer-grained segmentation and better adherence to weak and complex boundaries in medical images. For the j-th superpixel region $R_{j}$, the region-level uncertainty is computed as:

(2) $$u_{j}\left(x_{n}^{t}\right)=\frac{\sum_{i\in \Omega }I\left(i\epsilon R_{j}\right)E_{i}\left(x_{n}^{t}\right)}{\sum_{i\in \Omega }I\left(i\epsilon R_{j}\right)},$$

where $I\left(\cdot \right)$ denotes the indicator function.

Next, to reduce the influence of large low-entropy background regions, we filter the entropy values of the $N_{s}$ superpixel regions and extract regions associated with higher prediction uncertainty, which are typically concentrated in foreground and boundary areas. Specifically, we apply K-means++ [30] to adaptively partition the region-level uncertainties ${\left\{u_{j}\left(x_{n}^{t}\right)\right\}}_{j=1}^{N_{s}}$ into K=2 clusters by minimizing the following objective. By setting K=2, we divide the region-level uncertainties into two distinct clusters. The first is a high-uncertainty cluster, usually capturing challenging tumor boundaries or foregrounds. The second is a low-uncertainty cluster representing normal brain tissue and clear background. This clear division effectively isolates the most informative superpixel regions. The K-Means++ clustering is formulated as follows:

(3) $$J=\sum_{k=1}^{K}\sum_{u_{j}\in V_{k}}{\left\|u_{j}\left(x_{n}^{t}\right)-\mu_{k}\right\|}_{2}^{2},$$

(4) $$\mu_{k}=\frac{1}{\left|V_{k}\right|}\sum_{u_{j}\in C_{k}}u_{j}\left(x_{n}^{t}\right),$$

where ${\left\|\cdot \right\|}_{2}^{2}$ is the squared Euclidean distance, $\mu_{k}$ is the cluster $V_{k}$ centroid, $\left|V_{k}\right|$ is the number of regions assigned to cluster $V_{k}$. The cluster with the higher centroid value is designated as the high-uncertainty set $S_{h}$. We enforce $S_{h}\geq \beta \cdot N_{s}$ to ensure sufficient foreground coverage. The parameter β, set to 0.1 in our experiments, serves as a safety threshold to guarantee the minimum required ratio of foreground coverage. Finally, RUA uncertainty $U\left(x_{n}^{t}\right)$ is derived by a conditional aggregation:

(5) $$U\left(x_{n}^{t}\right)=\frac{\sum_{j=1}^{N}I\left(u_{j}\left(x_{n}^{t}\right)\in S_{h}\right)\cdot u_{j}\left(x_{n}^{t}\right)}{\sum_{j=1}^{N}I\left(u_{j}\left(x_{n}^{t}\right)\in S_{h}\right)+\vartheta },$$

where ϑ is a smoothing factor. Based on $U\left(x_{n}^{t}\right)$, we rank all unlabeled samples and select the top M samples to form the candidate set $D_{c}^{t}$ for the subsequent fine-grained selection. We set the size of the candidate set M to 5 times the annotation budget B. This setting not only effectively filters out a large number of low-informative samples, but also reserves sufficient headroom for optimization in the UGCS.

#### 2.2.2. Uncertainty-Guided Core-Set Selection

Following the coarse-grained screening via RUA, $D_{c}^{t}$ may still contain adjacent slices with high semantic similarity, leading to information redundancy. To mitigate this and optimize the annotation budget, we propose UGCS. Adapted from the Core-set framework [31], UGCS formulates sample selection as a greedy optimization problem that balances diversity and uncertainty of the samples.

Let S denote the set of currently selected samples, and $z_{n}=g\left(x_{n}^{t}\right)$ represent the deep feature vector of a candidate sample $x_{n}^{t}\epsilon D_{c}^{t}$ in the latent space, where $g\left(\cdot \right)$ is the encoder of the network. To quantify diversity, we define a distance metric $\delta \left(x_{n}^{t},S\right)$, which computes the minimum Euclidean distance from $x_{n}^{t}$ to the manifold of selected samples:

(6) $$\delta \left(x_{n}^{t},S\right)=\underset{x_{m}^{t}\in S}{\min}{\left\|z_{n}-z_{m}\right\|}_{2}^{2}.$$

A lower $\delta \left(x_{n}^{t},S\right)$ indicates that $x_{n}^{t}$ lies in a high-density region already covered by S (i.e., information redundancy). Conversely, a higher value implies that the sample occupies a sparse region in the latent feature space.

To integrate this diversity measure with RUA uncertainty $U\left(x_{n}^{t}\right)$, we normalize both scores to the range $\left[0,\;1\right]$ at each iteration:

(7) $$\tilde{\delta }\left(x_{n}^{t}\right)=\frac{\delta \left(x_{n}^{t},S\right)-\underset{k}{\min}\delta \left(x_{k}^{t},S\right)}{\underset{k}{\max}\delta \left(x_{k}^{t},S\right)-\underset{k}{\min}\delta \left(x_{k}^{t},S\right)},$$

(8) $$\tilde{U}\left(x_{n}^{t}\right)=\frac{U\left(x_{n}^{t}\right)-\underset{k}{\min}U\left(x_{k}^{t}\right)}{\underset{k}{\max}U\left(x_{k}^{t}\right)-\underset{k}{\min}U\left(x_{k}^{t}\right)},$$

where k iterates over all samples in $D_{c}^{t}$. Finally, the active selection is formulated as a greedy optimization problem.

For each iteration, the algorithm selects the next sample $x^{*}$ that maximizes the weighted combination of uncertainty and diversity:

(9) $$x^{*}=\underset{x_{i}\epsilon D_{c}^{t}\backslash S}{\mathrm{argmax}}\left[\lambda \cdot \tilde{\delta }\left(x_{n}^{t}\right)+\left(1-\lambda \right)\cdot \tilde{U}\left(x_{n}^{t}\right)\right],$$

(10) $$S\leftarrow S\cup \left\{x^{*}\right\},$$

where $\lambda \in \left[0,\;1\right]$ is a balancing coefficient, set to 0.5 in our experiments. It trades off sample uncertainty against diversity, preventing the algorithm from over-sampling redundant adjacent slices from the candidate pool. This selection process iterates until the size of S reaches the predefined annotation budget B. After the selection loop is completed, the final set S is designated as the labeled set $D_{l}^{t}$ and submitted to medical experts for manual annotation.

### 2.3. Segmentation Network Architecture

**Figure 2:**
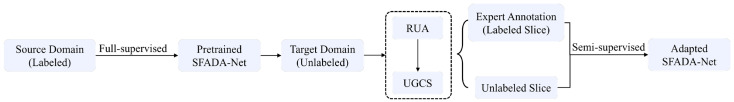
Complete flowchart of the SFADA framework.

We propose SFADA-Net for brain tumor segmentation in domain adaptation scenarios, and employ it as the core segmentation backbone of the entire SFADA framework. SFADA-Net not only performs the final segmentation task in the target domain, but also serves as a bridge between the active sample selection stage and the semi-supervised adaptation stage. During the selection phase (see Section 2.2), it provides pixel-wise predictive probability maps to the RUA module for uncertainty estimation and supplies deep latent representations to the UGCS module for diversity measurement. During the adaptation phase, SFADA-Net serves as the core model optimized under the semi-supervised learning (SSL) paradigm. A complete flowchart of the SFADA framework is shown in Figure 2.

SFADA-Net consists of three main components: a feature encoder composed of 4 SP-Mamba blocks, skip connections augmented with Residual Blocks, and a decoder based on transposed convolutions to predict the final segmentation mask. Each SP-Mamba block integrates the Dual-Path Multi-Kernel Convolution (DPMKC) and Structure-Aware Prompted Mamba (SAPM) modules, with channel numbers progressively increasing as 48, 96, 192, 384, and 768 to capture local details and long-range spatial dependencies at multiple scales. The overall architecture and key module designs are shown in Figure 2. Details of SFADA-Net are described below.

#### 2.3.1. Dual-Path Multi-Kernel Convolution Module

**Figure 3:**
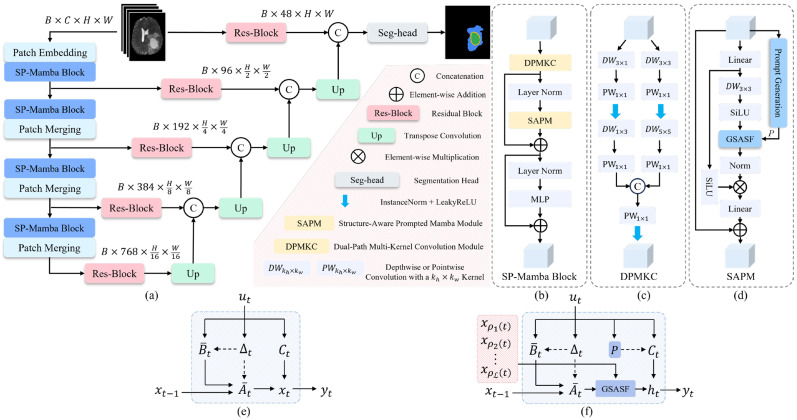
Overview of the proposed SFADA-Net, including the overall architecture and key module designs. (**a**) The complete U-shaped encoder–decoder framework. (**b**) Structure of the SP-Mamba block. (**c**) Detailed design of the Dual-Path Multi-Kernel Convolution (DPMKC) module. (**d**) Structure of the Structure-Aware Prompted Mamba (SAPM) module. (**e**) SSM in the standard Mamba and (**f**) SSM in the proposed SAPM module, where the residual term D is not displayed for simplicity.

The DPMKC is positioned before the designed SAPM module and is primarily responsible for extracting local detail information from features. As illustrated in Figure 3c, DPMKC uses a two-branch design that combines direction awareness and multi-scale aggregation. The first branch is the direction-aware path. It uses depth-wise separable convolutions with 3×1 and 1×3 kernels to model horizontal and vertical spatial dependencies. A point-wise convolution follows to fuse and interact features across scales. The second branch is the multi-scale aggregation path. It connects 3×3 and 5×5 convolutions in series to obtain local texture and wider contextual information. The output of the direction-aware path is computed as:

(11) $$F_{dir}={DW}_{1\times 3}\left({DW}_{3\times 1}\left(X\right)\right),$$

(12) $$F_{mul}={DW}_{5\times 5}\left({DW}_{3\times 3}\left(X\right)\right),$$

where $X\in R^{C_{h}\times H\times W}$ denotes the input feature map, ${DW}_{k_{h}\times k_{w}}$ represents a depth-wise separable convolution with a $k_{h}\times k_{w}$ kernel, followed by a point-wise convolution with a 1×1 kernel, instance normalization, and a Leaky ReLU activation. $F_{dir}$ denotes the output of the direction−aware pathway and $F_{mul}$ is denotes the output of the multi-scale aggregation pathway.

The two branch outputs are concatenated channel-wise and fused by a convolution block. A residual connection reuses the input features. The final output Y is:

(13) $$Y={Con}_{1\times 1}\left(\left[F_{dir}∥F_{mul}\right]\right)+X,$$

where $\left[\cdot ∥\cdot \right]$ is channel-wise concatenation and ${Con}_{1\times 1}$ is a 1×1 convolution followed by normalization and active function.

#### 2.3.2. Structure-Aware Prompted Mamba Module

The standard Mamba [32] is based on a state space model (SSM) with the discrete state transition equation and observation equations:

(14) $$x_{t}={\bar{A}}_{t}x_{t-1}+{\bar{B}}_{t}u_{t},$$

(15) $$y_{t}=C_{t}x_{t}+Du_{t},$$

where $x_{t}$ denotes the state variable, $u_{t}$ is the input sequence, and $y_{t}$ is the output sequence. Given a time step $\Delta_{t}$, the state transition matrix is ${\bar{A}}_{t}=exp\left(\Delta_{t}A\right)$, the input matrix is ${\bar{B}}_{t}={\left(\Delta_{t}A\right)}^{-1}\left(\exp\left(\Delta_{t}A\right)-I\right)\Delta_{t}B\approx \Delta_{t}B$. $C_{t}$ is the output matrix, and D is direct transmission matrix. In Mamba, ${\bar{A}}_{t}$, ${\bar{B}}_{t}$, $C_{t}$, and $\Delta_{t}$ are all functions of the input sequence $u_{t}$.

Although the standard Mamba effectively captures long-range dependencies in 1D sequential data, its application to 2D vision tasks introduces two critical limitations: First, flattening images via directional scanning inherently disrupts spatial adjacency and degrades local structural continuity [33]. Second, Mamba’s strictly causal formulation restricts pixels to attending only to preceding tokens, blinding the model to subsequent context and hindering global perception.

To mitigate the first issue, we adapt the Structure-Aware State Fusion (SASF) mechanism from Spatial-Mamba [34]. By integrating SASF between the state transition and observation equations, we establish neighborhood connectivity within the state space. SASF calculates a new structure-aware state variable $h_{t}$ by aggregating neighboring state variables in a 2D neighborhood:

(16) $$h_{t}=\sum_{l\in N}\alpha_{l}x_{\rho_{l}\left(t\right)},$$

where N denotes the 2D neighborhood set consisting of L state variables, $\alpha_{k}$ represents the learnable weight, and $\rho_{l}\left(t\right)$ is the index of the l-th neighboring state corresponding to position t. By fusing local neighborhood states, the structure-aware state variable $h_{t}$ incorporates both the global information from the standard Mamba and the local structural relationships.

To overcome the causal limitation, we exploit the mathematical parallels between state space models and attention mechanisms. Recognizing that $C_{t}$ essentially acts as a query matrix, we integrate a semantic prompt P into the observation equation [35].

Inspired by Spatial-Mamba [34] and Mamba-IRv2 [35], we extend the standard Mamba by incorporating structure-aware fusion and prompt enhancement, and propose the SAPM module, as shown in Figure 3d. Rather than redefining the Mamba framework, SAPM improves its performance through structure-aware and prompt-enhanced methods, as illustrated in Figure 3e,f. The core operations of SAPM are formulated by three key equations, namely the state transition equation, the grouped structure-aware state fusion (GSASF) equation, and the prompt-enhanced observation equation:

(17) $$x_{t}={\bar{A}}_{t}x_{t-1}+{\bar{B}}_{t}u_{t},$$

(18) $$h_{t}=\sum_{g=1}^{G}w_{t}^{g}\left(\sum_{i,j\in N_{d_{g}}}k_{ij}^{d_{g}}\cdot x_{t+iW+j}^{g}\right),$$

(19) $$y_{t}={(C}_{t}+P)h_{t}+Du_{t},$$

where $x_{t}$ is the original state variable, and $h_{t}$ denotes the structural perception state variable. Equation (18) is a practical implementation of the structure-aware formulation defined in Equation (16). The term $k_{ij}^{d_{g}}$ represents the weight of a 3×3 depth-wise filter at location $\left(i,j\right)$ in group g with dilation factor $d_{g}$. The neighborhood set is defined as $N_{d_{g}}=\left\{\left(i,j\right)|i,j\in \left\{-d_{g},0,d_{g}\right\}\right\}$. $x_{t+iW+j}^{g}$ refers to the neighboring states of $x_{t}^{g}$ within g, where W denotes the image width. The term $w_{t}^{g}$ is a pixel-wise learnable weight, and P is the semantic prompt vector.

In the GSASF equation, the state $x_{t}$ is divided into G=4 channel groups, each processed with a 3×3 depth-wise convolution with dilation factors $d_{g}\in \left\{1,3,5,7\right\}$. We then employ a pixel-adaptive weighting module to generate a weight map for each group. This module consists of *a* 1×1 convolution, a Leaky ReLU activation, followed by another 1×1 convolution and a Sigmoid activation:

(20) $$w_{t}^{g}=Sigmoid({Con}_{1\times 1}(\sigma ({Con}_{1\times 1}(x_{t}^{g})))).$$

These weight maps are normalized along the channel dimension and multiplied element-wise with their corresponding group features. Finally, the weighted group outputs are concatenated along the channel dimension to obtain $h_{t}$. In this way, local neighborhood information is introduced into the state representation while keeping the long-range modeling ability of Mamba.

To construct the prompt P, SAPM maintains a learnable prompt pool $E\in R^{N_{p}\times D_{p}}$, where $N_{p}$ denotes the length of tokens and $D_{p}$ is the state dimension. Given a flattened input feature map $x_{t}^{\prime }\in R^{L\times C_{h}}$, a routing network projects the channel dimension from $C_{h}$ to $N_{p}$ using a fully connected layer, followed by a LogSoftmax to produce normalized log-probabilities representing the likelihood of each prompt being selected for each spatial location.

We then apply the Gumbel–Softmax reparameterization [36] to obtain a differentiable discrete assignment, yielding a binary routing mask $M\in R^{L\times N_{p}}$. The prompt is computed as P=M⋅E and incorporated into $C_{t}$ via residual addition, forming a prompt-enhanced observation equation. This mechanism endows the model with attention-like behavior, enabling pixel-wise semantic querying across the image and alleviating perception gaps in unscanned regions [35].

### 2.4. Semi-Supervised Learning for Source-Free Active Domain Adaptation

To effectively leverage both the actively selected labeled target samples $D_{l}^{t}$ and the remaining unlabeled samples $\;D_{u}^{t}$, we employ a SSL framework based on the Mean Teacher paradigm [37]. The framework consists of a student model parameterized by $\theta_{s}$ and a teacher model parameterized by $\theta_{t}$, which share an identical architecture.

In the training phase, the student model parameters $\theta_{s}$ are optimized via backpropagation, whereas the teacher model parameters $\theta_{t}$ are updated using the exponential moving average (EMA) of the student parameters:

(21) $$\theta_{t}^{\prime }=\alpha \theta_{t}+\left(1-\alpha \right)\theta_{s},$$

where α is the smoothing coefficient, set to 0.996.

We apply distinct data augmentation strategies to construct a consistency regularization constraint. For the labeled set $D_{l}^{t}$, the student model is trained using standard supervised learning. The supervised loss $L_{s}$ is defined as the summation of the Cross-Entropy loss and the Dice loss:

(22) $$L_{s}=-\frac{1}{\left|\Omega \right|}\sum_{i\in \Omega }\sum_{c=1}^{C}y_{i,c}\log\left(p_{s,i,c}\right)-\frac{1}{C}\sum_{c=1}^{C}\frac{2\sum_{i\in \Omega }p_{s,i,c}y_{i,c}}{\sum_{i\in \Omega }p_{s,i,c}+\sum_{i\in \Omega }y_{i,c}},$$

where $y_{i,c}$ is the one-hot ground truth (GT) label, and $p_{s,i,c}$ is the student’s predicted probability for class c at pixel i.

For the unlabeled data $D_{u}^{t}$, weak augmentation is applied to the teacher input to generate pseudo-labels, while CutMix [38] is employed for data augmentation on the student input. To mitigate the noise in pseudo-labels, we apply a confidence thresholding mechanism. The unsupervised consistency loss $L_{u}$ is computed as the pixel-wise cross-entropy between the student’s prediction on the strongly augmented view and the teacher’s high-confidence pseudo-labels:

(23) $$L_{u}=-\frac{1}{\left|\Omega \right|}\sum_{i\in \Omega }I\left(\underset{c}{\max}p_{t,i,c}>\tau \right)\sum_{c=1}^{C}{\hat{y}}_{t,i,c}\log\left(p_{s,i,c}^{aug}\right),$$

where $I\left(\cdot \right)$ is the indicator function that selects pixels where the teacher’s maximum probability exceeds the confidence threshold τ, ${\hat{y}}_{t,i,c}$ is the hard pseudo-label derived from the teacher, and $p_{s,i,c}^{aug}$ is the student’s prediction under CutMix augmentation.

The total loss function is formulated as:

(24) $$L={\gamma_{1}L}_{s}+\gamma_{2}L_{u},$$

where $\gamma_{1}$ is set to 2 in our experiments. $\gamma_{2}$ is initialized to 0 and kept at 0 for the first 200 iterations, and then linearly increased from 0.5 to 1.0 over iterations 200–400 to gradually incorporate the unsupervised loss.

## 3. Experiments and Results

### 3.1. Dataset

To evaluate the effectiveness and generalizability of our RUGS strategy and SFADA-Net, we employed four diverse medical imaging datasets: BraTS-2021 [39,40], BraTS-SSA 2023 [41], BraTS-PED 2023 [42], and BraTS-MEN 2023 [43]. Each case consists of four MRI modalities: T1-weighted (T1), contrast-enhanced T1-weighted (T1CE), T2-weighted (T2), and T2-weighted fluid attenuated inversion recovery (T2-FLAIR).

- BraTS-2021: This dataset comprises 1251 adult glioma cases annotated by experienced radiologists.
- BraTS-SSA 2023: Specifically gathered from Sub-Saharan Africa, this dataset contains 60 annotated cases. It is included to assess model performance under the domain shift caused by population differences and data scarcity in the African region.
- BraTS-PED 2023: This dataset consists of 99 pediatric glioma cases. It evaluates the model’s ability to handle the differences between pediatric and adult tumor anatomy.
- BraTS-MEN 2023: Focusing on adult intracranial meningioma, this dataset introduces a distinct tumor type. We randomly sampled 100 annotated cases from this collection for training and testing.

### 3.2. Model Training and Implementation Details

Our framework was implemented in PyTorch 2.0.1 on an NVIDIA RTX 3090 GPU, with input images cropped to 224×224 patches. The training procedure involved two stages: source domain pre-training and domain adaptation. In both stages, the model was optimized using SGD, with the learning rate updated via a Poly scheduler with power 0.9.

Regarding data preprocessing, all MRI scans were co-registered to the same anatomical template, resampled to an isotropic resolution of 1 × 1 × 1 mm^3^, and skull-stripped. To mitigate intensity variations across different patients and scanners, each MRI modality was independently normalized using Z-score standardization based on the non-zero brain regions. As the datasets utilized in our experiments provided complete multi-modal data for all cases, specific mechanisms for handling missing modalities were not required in this study. To mitigate overfitting and enhance model robustness during model training, we adopted a diverse set of data augmentation techniques, including spatial transformations, Gaussian noise, contrast adjustments and axis mirroring.

In the pre-training stage, the source domain dataset was randomly split into training, validation, and testing sets with a ratio of 7:1:2. The model was trained in a fully supervised manner for 300 epochs with a batch size of 32 and an initial learning rate of 0.01, using the supervised loss $L_{s}$.

For the adaptation stage, we first employed the RUGS strategy to select valuable samples. To simulate clinical annotation scenarios, we accessed target labels solely during this phase, with a labeling budget of 5%. The RUGS hyperparameters β, λ, $N_{s}$ and the capacity of $D_{c}^{t}$ were set to 0.1, 0.5, 400, and five times the budget, respectively. Subsequent training utilized these labeled samples in the SSL framework described in Section 2.4. The model was trained for 20k iterations with a batch size of 16 (4 labeled, 12 unlabeled) and an initial learning rate of 0.001. We adopted 5-fold cross-validation and an early stopping mechanism with a patience of 10.

In the inference phase, we deployed our 2D network to 3D MRI volumes in a slice-by-slice manner, and subsequently reconstructed the 3D volumes by stacking the resulting 2D predictions along the longitudinal axis. Although 2D inference inherently lacks inter-slice constraints, we deliberately omitted all 3D post-processing steps to isolate and rigorously evaluate the intrinsic baseline performance of the SFADA framework.

### 3.3. Comparison with State-of-the-Art Methods

To quantitatively assess the accuracy of the tumor segmentation results, we employed four metrics: Dice Similarity Coefficient (DSC), Sensitivity, Precision, and the 95th percentile Hausdorff distance (HD95). For the four datasets, we calculated the average quantitative results of each method for three tumor sub-regions: Whole Tumor (WT), Tumor Core (TC), and Enhancing Tumor (ET). For each metric, both the mean value and the standard deviation are reported. The standard deviation was computed based on the metric values of all individual test samples from a single experimental run, reflecting the performance variability across cases.

#### 3.3.1. Quantitative Comparison of Backbones on the Source Domain

Given that the BraTS-2021 dataset contains the most extensive patient data and annotations, we selected it as the source domain for model pre-training. To demonstrate the improved performance of SFADA-Net, we benchmarked it against several state-of-the-art (SOTA) segmentation models, including U-Net [12], UNETR [44], SwinUNETR-V2 [45], TransAttUnet [9], VM-UNet [11], and Swin-UMamba [13].

**Figure 4:**
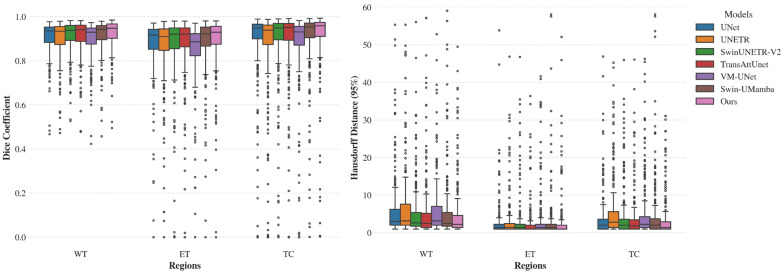
Boxplot comparison of segmentation performance achieved by different backbone models on the source domain (BraTS-2021) dataset. Left panel: Dice coefficients for whole tumor (WT), enhancing tumor (ET), and tumor core (TC) regions. Right panel: 95% Hausdorff distance (HD95) for the same regions. Each boxplot displays the median (center line), interquartile range (IQR, box), whiskers extending to 1.5 times the IQR, and outliers (points).

As shown in Table 1, SFADA-Net outperforms competing models across multiple metrics. Notably, it surpasses U-Net with DSC improvements of 1.79% and 2.06% for WT and TC, respectively. Furthermore, the lower HD95 scores demonstrate its superior boundary delineation precision. Figure 4 further visualizes the full per-case distribution of DSC and HD95, clearly demonstrating the superior median performance, smaller variance, and fewer outliers of our SFADA-Net even on the source domain.

**Figure 5:**
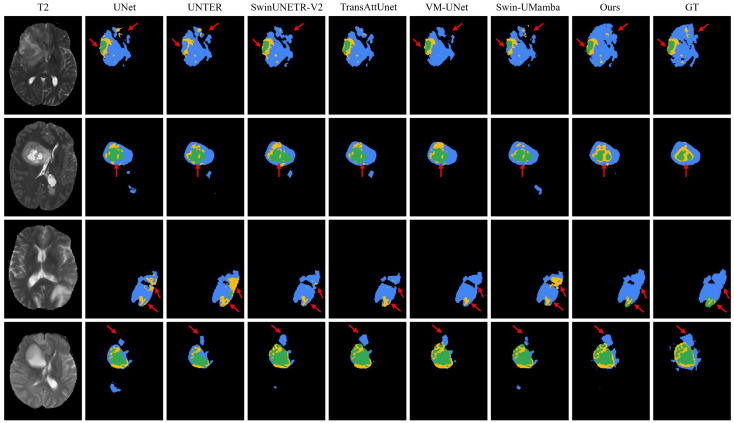
Visualization of brain tumor segmentation results by different methods on the BraTS-2021 dataset. Rows represent different patient cases. Tumor regions are colored as follows: edema (ED, blue), enhancing tumor (ET, yellow), and necrotic/non-enhancing tumor cores (NCR/NET, green). Red arrows indicate significant differences.

Figure 5 illustrates qualitative brain tumor segmentation results of different methods on the BraTS-2021 dataset. Each row represents a different patient case, with the original MRI scan displayed in the leftmost column. This performance advantage can be primarily attributed to the sample selection mechanism rather than the subsequent training strategy. Our proposed method achieves clearer delineation of tumor subregions and higher overlap with the ground truth, particularly in the arrow-marked areas. In these areas, it successfully recovers finer tumor details and subtle structures (such as small enhancing components and precise boundaries) that are frequently missed or inaccurately segmented by other approaches.

#### 3.3.2. Quantitative Comparison of Adaptation Strategies with SFADA-Net

To assess the effectiveness of the proposed RUGS strategy, we compare it with two SOTA SFDA methods, DPL [21] and UPL [22]. For active sample selection, we evaluate five strategies under an identical annotation budget: (1) Random: selects samples randomly; (2) LC [46]: selects samples with the least confidence; (3) Core-set [31]: selects samples based on a set-cover problem; (4) STDR [25]: selects samples with maximal and minimal distribution shifts from the source and (5) UGTST [24]: selects samples based on global uncertainty and diversity.

Furthermore, we incorporate two representative SSL methods for comparison: (1) URPC [47]: enforces multi-scale prediction consistency via a pyramid network to exploit unlabeled data; and (2) ABD [48]: employs a confidence-guided bidirectional displacement module to suppress unreliable regions and generate complementary training samples. Both baselines were trained on the same randomly selected labeled subset.

Finally, we include three baselines to establish performance bounds: (1) Source only: directly uses the model pre-trained in the source domain for inference in the target domain, serving as the lower bound; (2) Target only: trains a model from scratch using labeled target data without source pre-training; and (3) Fine-tune: fine-tunes the pretrained source model using the fully annotated target training set based on supervised learning.

To ensure a fair comparison, all methods are implemented using the SFADA-Net backbone and evaluated via 5-fold cross-validation. Quantitative results for the BraTS-SSA, PED, and MEN datasets are summarized in Table 2, Table 3 and Table 4, respectively.

On the BraTS-SSA 2023 dataset (Table 2), RUGS achieves the highest DSC among all compared methods. Compared with conventional active learning strategies including LC and Core-set, RUGS leverages region-level uncertainty and UGCS to effectively avoid redundant sample selection. Compared with the SSL methods (URPC and ABD), our method selects more informative samples for annotation, enabling the fine-tuned model to achieve superior brain tumor segmentation and higher segmentation accuracy. Notably, our method’s performance closely approaches full fine-tuning, reducing the supervision gap to within 0.12–1.02% DSC while demonstrating robust boundary delineation with consistently low HD95 across all regions.

On the BraTS-PED 2023 dataset (Table 3), RUGS maintained an advantage over other methods. While “Source only” suffered a substantial drop in the TC region (40.94%), RUGS restored the TC DSC to 84.10%, surpassing the Core-set (82.76%) and UGTST (82.11%) strategies. This significant performance recovery demonstrates the capability of RUGS to accommodate the anatomical discrepancies between pediatric and adult brain tumors. RUGS effectively captures the core morphological alterations of pediatric tumors, which are the key factors accounting for the failure of “Source only”. Similarly, in the ET region, RUGS achieved the highest score of 58.42%. The method also demonstrated superior boundary control, recording an HD95 of 12.10 mm for TC, which outperformed SFDA methods, positioning it closest to “Fine-tune”.

On the more challenging BraTS-MEN 2023 dataset (Table 4), performance gaps between different methods became more pronounced. The significant difference between “Source only” and “Fine-tune” indicates a substantial domain shift from the BraTS-2021 to the BraTS-MEN dataset. Meningiomas are fundamentally distinct from gliomas in terms of structural and signal intensity characteristics, which leads to performance degradation of baseline domain adaptation strategies. However, RUGS achieved DSC of 76.23%, 88.57%, and 78.87% for WT, TC, and ET, respectively, all higher than those of other SOTA methods, with a particularly notable advantage in the WT region. In terms of boundary accuracy, RUGS also yielded lower HD95, with 29.80 mm, 18.40 mm, and 27.45 mm for WT, TC, and ET.

**Figure 6:**
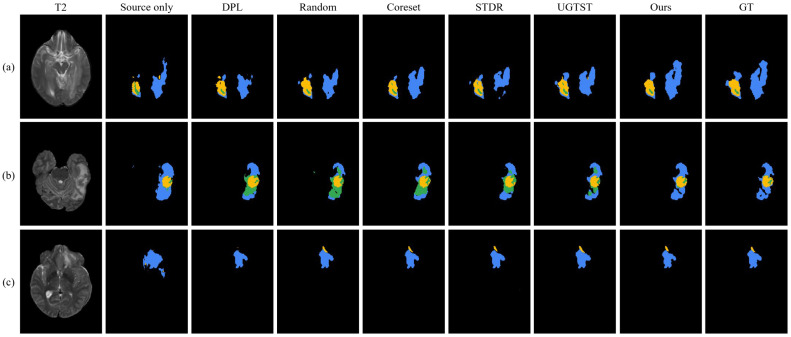
Visualization of brain tumor segmentation results by different methods. (**a**) Samples from BraTS-SSA 2023, (**b**) samples from BraTS-PED 2023, and (**c**) samples from BraTS-MEN 2023. Tumor regions are colored as follows: NCR/NET is in green, ET is shown in yellow, and ED is in blue.

Figure 6 provides visual comparisons of brain tumor segmentation results across the three datasets. Our method consistently outputs maps that closely align with the ground truth, offering sharper boundary delineation and accurate subregion identification. For the whole tumor, our approach ensures complete spatial coverage, effectively mitigating the boundary discontinuities and irregular artifacts prevalent in competing methods. Within the NCR/NET and ED, it generates structurally cohesive predictions with notably fewer scattered false positives. Furthermore, our model successfully preserves the fine details of small or highly irregular lesions, which are often over-smoothed or entirely missed by the baselines.

#### 3.3.3. Quantitative Comparison of Backbones Under RUGS on Target Domain

To further investigate the impact of the network architecture on domain adaptation performance, we compared SFADA-Net with several SOTA segmentation models on the BraTS-SSA 2023 dataset. To ensure a fair and controlled comparison, all competing backbones—including UNet, UNETR, SwinUNETR-V2, TransAttUnet, VM-UNet, and Swin-UMamba—were trained using the proposed RUGS active learning strategy under the same annotation budget. The quantitative results are summarized in Table 5. The results indicate that SFADA-Net consistently outperforms the other models across all metrics. Our method exhibits superior adaptability to domain shifts, yielding more accurate tumor delineation and better boundary preservation compared to other models.

### 3.4. Ablation Studies

#### 3.4.1. Impact of Sample Selection Parameters

**Figure 7:**
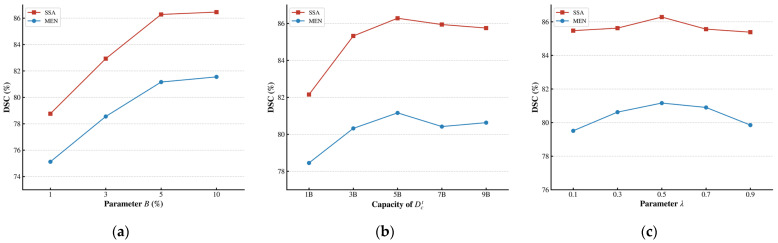
Impact of sample selection parameters on BraTS-SSA 2023 and BraTS-MEN 2023 dataset. The reported DSC represents the average score of WT, TC, and ET regions. (**a**) Effect of B on segmentation accuracy. (**b**) Influence of the capacity of $D_{c}^{t}$ with B=5%. (**c**) Impact of the UGCS parameter λ.

We first evaluate the impact of B from 1% to 10% on the model using the BraTS-SSA 2023 and BraTS-MEN 2023 dataset, as depicted in Figure 7a, where B denotes the percentage of target-domain data manually annotated. Increasing B initially improves segmentation accuracy, but the improvement becomes limited as B gets larger. Specifically, increasing B from 5% to 10% doubles the annotation cost but results in very little performance gain. To achieve a favorable trade-off between accuracy and labeling cost, we employ B at 5% for all other experiments.

Moreover, we examine the influence of the capacity of $D_{c}^{t}$ selected by RUA. As shown in Figure 7b, while a larger candidate pool initially benefits the model by offering higher diversity, performance drops when the size becomes excessive. We attribute this to the introduction of noisy or redundant instances within an overly large pool. Thus, we set the candidate set capacity $D_{c}^{t}$ to five times B.

Finally, we assess the influence of λ in UGCS, which regulates the uncertainty-diversity trade-off. As presented in Figure 7c, smaller λ overemphasize uncertainty and provide only modest gains. Model performance peaks at λ = 0.5 across the two target domains, while further increasing λ leads to a performance decline. Based on these observations, we set λ = 0.5 for all other experiments.

#### 3.4.2. Impact of Key Components

The SFADA-Net model is built upon a UNet-like segmentation architecture [12] and integrates the SAPM and DPMKC modules. We conducted ablation experiments on the BraTS-2021 dataset to evaluate each component (Table 6). Incorporating Mamba, SAPM, or DPMKC individually into the baseline consistently improves segmentation metrics. Specifically, the SAPM and DPMKC modules provide more distinct performance gains compared to the SSM alone. Most notably, the simultaneous integration of both SAPM and DPMKC yields the most significant boost in DSC and a substantial reduction in HD95 across all tumor subregions, outperforming all single-module configurations. This confirms that the proposed modules work synergistically to maximize the overall segmentation accuracy.

Table 7 compares the performance of different models in terms of complexity and computational efficiency, specifically evaluating the parameter count, floating-point operations (FLOPs), and inference time across current mainstream architectures and our ablation configurations. The experimental results show that our proposed model maintains a relatively compact scale, with fewer parameters than several mainstream Transformer-based and Mamba-based networks. In terms of computational cost, our method also maintains a moderate level of FLOPs without excessive demand. Although the introduction of the SAPM and DPMKC modules increases inference time and computational overhead compared to the baseline model, the complete model still achieves faster inference speed than other Mamba-based architectures. Overall, the experiment validates that SFADA-Net improves segmentation performance without excessively consuming computational resources.

The ablation study results summarized in Table 8 demonstrate the consistent performance gains achieved by integrating the RUGS strategy components on the BraTS-MEN 2023 dataset. Starting from the baseline (B), which relies solely on pre-trained pseudo-labels, the introduction of RUA provides a more robust selection mechanism than the conventional Entropy-based method, notably elevating the WT DSC from 68.15% to 72.85% while simultaneously reducing the HD95. Furthermore, the full configuration incorporating UGCS proves more effective than the Core-set strategy; while Core-set offers competitive sensitivity, UGCS achieves substantially better boundary delineation and precision.

## 4. Discussion

Quantitative results in Table 2, Table 3 and Table 4 indicate that SFADA-Net, combined with RUGS, outperforms existing SFDA and SFADA methods across multiple target domains (BraTS-SSA, BraTS-PED, and BraTS-MEN). Our method achieves segmentation performance comparable to fully supervised training while using only 5% of annotated data. This effectiveness stems from the combined sample selection strategy and network architecture. Within RUGS, RUA filters out most low-entropy background regions and aggregates pixel-level uncertainty into semantically meaningful regions. UGCS balances sample diversity and uncertainty, reducing redundancy in purely uncertainty-based selection. This performance advantage can be primarily attributed to the sample selection mechanism rather than the subsequent training strategy. While semi-supervised methods such as URPC and ABD incorporate sophisticated consistency regularization or pseudo-label refinement techniques, their effectiveness is inherently limited by the quality and representativeness of the initially labeled samples. As shown in Table 2, Table 3 and Table 4, even when equipped with advanced semi-supervised learning modules, these methods underperform compared to our approach, which emphasizes the selection of informative and diverse samples prior to adaptation. What this really means is that, under low-annotation budgets, it is not the complexity of the training objective but the strategic selection of samples that makes the difference.

Moreover, SFADA-Net surpasses SOTA networks in both source and target domains (Table 1 and Table 5). The SAPM module captures long-range dependencies with linear complexity, crucial for delineating complex brain tumor structures. Specifically, the GSASF equation captures neighborhood structural information, and the prompt mechanism enhances querying semantic information. The DPMKC module complements Mamba by preserving local details, ensuring global consistency does not compromise local precision. This synergy is particularly beneficial for MRI with variable tumor textures.

In clinical settings, privacy regulations often hinder cross-center data sharing. Our source-free setting addresses this by using only source-pretrained weights and unlabeled target samples, eliminating source data access. Experiments confirm this framework effectively handles domain shifts caused by variations in equipment, demographics, and pathology. Furthermore, by actively selecting a small subset of target samples for annotation, we mitigate pseudo-label errors in unsupervised methods and achieve more reliable domain adaptation.

**Figure 8:**
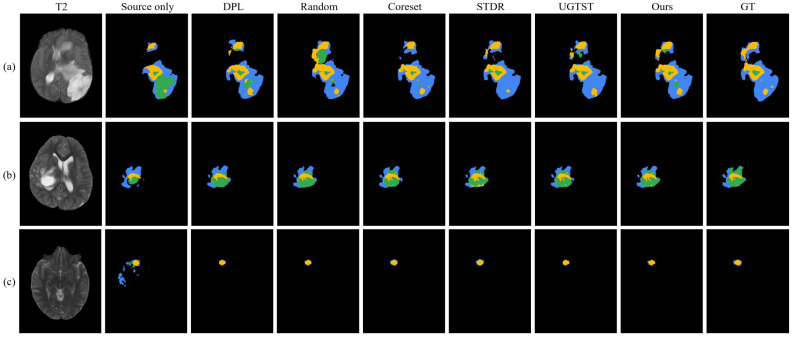
Visualization of representative failure cases of the proposed SFADA framework. (**a**) Samples from BraTS-SSA 2023, (**b**) samples from BraTS-PED 2023, and (**c**) samples from BraTS-MEN 2023. Tumor regions are colored as follows: NCR/NET is in green, ET is shown in yellow, and ED is in blue.

Although the proposed SFADA method outperforms SOTA methods on the majority of target-domain samples, it still has performance bottlenecks in some challenging samples. Figure 8 shows representative failure cases from the three target domains. In the top row, the WT and TC are generally well-delineated, under-segmentation is still observed around the boundaries between the ED region (blue) and the ET region (yellow). In the middle row, confusion between partial ED regions and NCR/NET regions (green) is observed, with the model misclassifying the green regions. In the bottom row (cases with extremely small, low-contrast lesions), the proposed method successfully detects ED regions missed by multiple baseline methods, yet there remains the problem of incomplete coverage of tumor regions.

Beyond the aforementioned failure cases and performance bottlenecks in challenging clinical scenarios, the proposed SFADA framework has several limitations. First, the current implementation operates on 2D slices to mitigate memory constraints, disregarding the volumetric context of MRI. Future work will extend the framework to 3D to fully leverage inter-slice correlations. Second, RUGS relies on the initial pre-trained model quality; significant domain shifts may compromise uncertainty estimation reliability. Finally, validation was restricted to brain tumor segmentation. Future studies will evaluate the proposed method’s robustness across broader medical imaging tasks.

## 5. Conclusions

In this study, we presented a novel SFADA framework for cross-center brain tumor segmentation. To address the challenges of data privacy, domain shift, and high annotation costs, we introduced the RUGS strategy. By aggregating uncertainty over multiple superpixel regions and leveraging Core-set selection, RUGS efficiently selects both informative and representative samples for annotation. Furthermore, SFADA-Net synergizes local feature extraction with long-range dependency modeling by integrating DPMKC and the SAPM module. Extensive validation on four multi-modal MRI datasets demonstrates that our approach consistently outperforms SOTA domain adaptation and active learning methods, achieving high segmentation accuracy under a limited annotation budget and approaching fully supervised performance. In terms of clinical implementation, our method effectively reduces the manual annotation workload for radiologists, enables rapid, low-cost deployment of AI-assisted diagnosis models in compliance with data privacy regulations, and facilitates the clinical translation of domain adaptation approaches.

Nevertheless, this study has several limitations. 2D slice-wise independent processing may overlook inter-slice contextual information in 3D MRI volumes. In addition, RUGS depends on source-pretrained model quality, with degraded uncertainty estimation under large domain shifts. Finally, the evaluation is limited to brain tumor segmentation tasks. Future work will extend the framework to full 3D volumetric processing, develop more robust uncertainty estimation techniques for large domain gaps, and validate the method on additional medical imaging tasks and modalities to further enhance its clinical applicability.

## Figures and Tables

**Table 1:** Quantitative comparison of segmentation methods on the source domain (BraTS-2021). Results are presented as mean ± standard deviation.

Method	DSC (%)			HD95 (mm)			Sensitivity (%)			Precision (%)		
	WT	TC	ET	WT	TC	ET	WT	TC	ET	WT	TC	ET
UNet [12]	90.44 ± 7.91	88.55 ± 16.97	86.73 ± 14.50	7.56 ± 13.13	4.74 ± 9.87	3.97 ± 11.08	89.41 ± 11.95	89.36 ± 17.42	87.37 ± 16.30	92.69 ± 5.95	90.18 ± 15.90	88.23 ± 13.19
UNETR [44]	90.29 ± 8.53	86.95 ± 18.14	85.94 ± 15.59	8.60 ± 14.89	7.64 ± 13.88	4.33 ± 10.52	89.44 ± 11.55	87.20 ± 18.32	85.81 ± 16.96	92.33 ± 7.85	89.20 ± 16.92	88.54 ± 13.89
SwinUNETR-V2 [45]	91.15 ± 7.57	88.52 ± 17.94	87.19 ± 14.78	6.48 ± 11.32	5.45 ± 11.16	3.68 ± 8.62	90.07 ± 11.24	88.61 ± 18.52	87.46 ± 16.16	93.23 ± 6.15	91.06 ± 15.93	88.98 ± 13.59
TransAttUnet [9]	91.18 ± 8.03	89.05 ± 16.22	87.05 ± 15.06	6.02 ± 10.51	4.59 ± 9.44	3.29 ± 8.81	89.32 ± 12.00	89.38 ± 16.73	86.97 ± 16.77	94.32 ± 5.63	91.13 ± 14.87	89.54 ± 13.26
VM-UNet [11]	89.60 ± 8.32	86.98 ± 17.87	84.30 ± 14.84	7.20 ± 11.62	5.72 ± 9.47	3.76 ± 7.54	88.87 ± 12.31	87.81 ± 18.80	85.99 ± 16.25	91.68 ± 6.83	88.94 ± 15.55	84.61 ± 14.28
Swin-UMamba [13]	91.41 ± 7.62	89.76 ± 15.63	87.90 ± 13.43	6.28 ± 10.95	5.50 ± 11.09	4.02 ± 10.81	90.90 ± 11.42	90.19 ± 16.33	88.40 ± 14.91	93.05 ± 5.97	91.56 ± 13.44	89.23 ± 12.60
SFADA-Net (Ours)	92.23 ± 7.04	90.61 ± 15.46	88.73 ± 13.11	5.73 ± 9.81	3.96 ± 6.93	2.95 ± 6.12	91.42 ± 10.03	91.30 ± 15.20	89.56 ± 14.62	93.91 ± 6.38	92.23 ± 12.56	89.69 ± 11.73

**Table 2:** Quantitative comparison of different methods on the BraTS-SSA 2023 dataset. Results are presented as mean ± standard deviation.

Method	DSC (%)			HD95 (mm)			Sensitivity (%)			Precision (%)		
	WT	TC	ET	WT	TC	ET	WT	TC	ET	WT	TC	ET
URPC [47]	88.46 ± 2.17	86.40 ± 3.54	79.14 ± 4.59	11.99 ± 3.80	9.92 ± 3.21	10.92 ± 2.49	87.70 ± 4.34	86.54 ± 3.41	79.45 ± 4.72	91.69 ± 3.56	88.19 ± 3.44	82.54 ± 4.35
ABD [48]	89.24 ± 2.48	85.14 ± 4.52	79.93 ± 3.67	11.77 ± 4.06	12.80 ± 6.18	11.36 ± 4.90	87.81 ± 4.08	86.35 ± 3.66	80.46 ± 4.79	91.66 ± 3.05	87.49 ± 4.73	83.58 ± 2.67
DPL [21]	87.31 ± 2.87	81.79 ± 3.81	73.80 ± 5.32	14.59 ± 3.99	11.80 ± 3.17	10.55 ± 2.95	88.33 ± 6.74	81.47 ± 3.45	68.17 ± 4.41	87.58 ± 5.80	86.28 ± 4.13	85.56 ± 4.93
UPL [22]	87.92 ± 2.55	83.45 ± 3.42	76.12 ± 4.81	12.15 ± 3.54	12.07 ± 2.98	10.92 ± 2.76	88.10 ± 6.12	84.25 ± 3.21	73.68 ± 4.05	88.85 ± 5.42	87.12 ± 3.86	86.40 ± 4.55
Source only	85.29 ±15.78	69.91 ± 28.68	65.76 ± 23.88	14.83 ± 19.20	19.08 ± 27.25	12.70 ± 19.15	85.36 ± 15.57	68.75 ± 27.51	57.88 ± 24.54	86.16 ± 17.42	76.29 ± 32.42	84.34 ± 23.90
Target only	85.75 ± 1.96	81.42 ± 4.09	74.89 ± 3.91	17.38 ± 4.57	12.70 ± 5.40	13.61 ± 2.21	86.38 ± 4.30	80.88 ± 3.83	75.56 ± 2.59	86.83 ± 2.41	85.50 ± 4.84	77.60 ± 5.05
Fine-tune	90.33 ± 2.31	87.93 ± 4.37	82.40 ± 3.75	9.90 ± 2.68	8.35 ± 3.31	9.15 ± 3.95	89.58 ± 3.03	87.81 ± 3.86	83.08 ± 4.92	92.15 ± 2.33	90.56 ± 4.73	84.31 ± 4.15
Random	88.23 ± 2.59	84.85 ± 4.27	79.69 ± 3.77	10.66 ± 1.74	13.64 ± 4.35	15.42 ± 2.40	87.06 ± 3.92	86.12 ± 4.35	81.27 ± 2.40	90.02 ± 3.32	86.16 ± 5.30	80.14 ± 5.63
LC [46]	88.42 ± 2.65	82.44 ± 5.39	78.03 ± 5.12	13.32 ± 3.23	16.18 ± 4.57	14.70 ± 4.12	86.40 ± 4.43	85.56 ± 3.83	80.52 ± 4.45	92.07 ± 2.38	83.62 ± 7.47	79.39 ± 4.21
Core-set [31]	88.82 ± 2.72	86.65 ± 4.13	80.31 ± 3.70	11.75 ± 2.86	8.64 ± 3.04	8.99 ± 4.10	88.00 ± 3.59	84.54 ± 5.43	78.13 ± 6.84	90.20 ± 3.70	90.69 ± 4.42	82.86 ± 5.90
STDR [25]	89.31 ± 2.94	86.78 ± 3.87	79.74 ± 4.34	11.55 ± 3.08	10.89 ± 3.05	11.34 ± 1.59	88.32 ± 3.39	87.59 ± 1.61	80.56 ± 2.59	90.96 ± 3.65	88.74 ± 3.71	80.95 ± 4.64
UGTST [24]	89.47 ± 2.61	86.58 ± 3.81	80.91 ± 3.21	13.68 ± 3.76	13.81 ± 4.31	12.28 ± 5.19	88.83 ± 3.68	86.50 ± 3.97	79.65 ± 5.07	90.72 ± 2.51	88.41 ± 3.88	84.20 ± 2.89
RUGS (Ours)	90.21 ± 1.96	86.91 ± 4.72	82.16 ± 3.24	10.42 ± 2.66	8.91 ± 3.24	9.85 ± 3.86	89.17 ± 3.57	87.77 ± 4.89	82.40 ± 5.28	92.41 ± 1.73	89.48 ± 4.61	83.03 ± 3.48

**Table 3:** Quantitative comparison of different methods on the BraTS-PED 2023 dataset. Results are presented as mean ± standard deviation.

Method	DSC (%)			HD95 (mm)			Sensitivity (%)			Precision (%)		
	WT	TC	ET	WT	TC	ET	WT	TC	ET	WT	TC	ET
URPC [47]	84.28 ± 2.38	81.80 ± 2.99	57.48 ± 15.09	16.04 ± 5.26	15.02 ± 4.48	12.74 ± 6.33	82.94 ± 3.71	79.24 ± 3.37	60.20 ± 13.87	88.97 ± 0.75	85.58 ± 1.53	60.81 ± 11.72
ABD [48]	85.43 ± 1.46	82.91 ± 2.00	57.55 ± 12.71	15.96 ± 8.40	12.67 ± 3.57	14.87 ± 5.98	84.29 ± 1.40	81.80 ± 1.75	58.61 ± 13.52	88.69 ± 1.97	86.44 ± 2.40	61.10 ± 10.81
DPL [21]	81.73 ± 2.74	74.98 ± 4.09	53.81 ± 14.03	17.58 ± 7.07	18.88 ± 6.46	15.13 ± 4.86	77.70 ± 4.33	70.20 ± 4.60	56.47 ± 13.87	89.73 ± 2.00	85.85 ± 3.07	60.76 ± 10.80
UPL [22]	82.45 ± 2.21	75.62 ± 3.68	54.33 ± 13.54	16.95 ± 6.85	19.12 ± 5.92	14.50 ± 5.10	78.55 ± 3.52	71.10 ± 4.18	56.12 ± 13.72	89.15 ± 1.96	86.20 ± 2.63	62.45 ± 11.36
Source only	77.81 ± 20.82	40.94 ± 31.75	44.45 ± 37.54	16.72 ± 19.19	22.08 ± 23.22	31.49 ± 39.58	75.80 ± 23.73	32.15 ± 29.88	43.53 ± 38.26	85.47 ± 18.81	86.07 ± 29.35	53.61 ± 40.68
Target only	83.61 ± 1.41	79.13 ± 2.29	54.52 ± 13.45	15.92 ± 1.16	17.01 ± 1.27	19.72 ± 8.22	82.42 ± 0.62	78.93 ± 1.99	56.15 ± 14.42	87.46 ± 2.57	82.47 ± 3.41	58.02 ± 14.07
Fine-tune	86.87 ± 1.78	84.17 ± 2.91	59.50 ± 12.78	11.55 ± 3.69	11.28 ± 3.57	13.57 ± 5.69	85.43 ± 2.69	82.41 ± 4.83	61.31 ± 11.12	90.12 ± 2.73	88.66 ± 3.27	63.85 ± 12.03
Random	84.10 ± 1.46	81.57 ± 2.03	57.05 ± 12.73	15.31 ± 4.54	15.47 ± 4.24	13.62 ± 6.94	82.61 ± 2.69	80.83 ± 0.93	59.23 ± 12.55	88.15 ± 2.06	84.81 ± 2.55	61.26 ± 10.72
LC [46]	80.43 ± 2.90	76.29 ± 2.99	55.14 ± 11.82	17.23 ± 4.27	16.82 ± 2.33	19.25 ± 6.40	76.90 ± 3.98	73.53 ± 3.16	56.68 ± 12.48	89.15 ± 2.07	84.88 ± 3.90	59.55 ± 11.20
Core-set [31]	85.67 ± 1.08	82.76 ± 2.82	58.01 ± 12.90	12.69 ± 2.15	13.30 ± 3.78	13.13 ± 6.53	83.68 ± 0.74	81.99 ± 1.86	58.64 ± 9.28	89.29 ± 2.97	85.39 ± 4.46	63.40 ± 13.93
STDR [25]	84.46 ± 2.14	81.93 ± 2.79	57.35 ± 11.40	15.88 ± 1.23	15.33 ± 3.25	16.51 ± 6.68	82.72 ± 2.65	81.10 ± 2.83	59.02 ± 10.82	88.81 ± 2.94	85.40 ± 3.87	61.16 ± 11.71
UGTST [24]	84.92 ± 1.67	82.11 ± 2.44	57.12 ± 14.04	16.82 ± 3.21	18.87 ± 2.68	17.20 ± 7.97	83.48 ± 2.34	82.08 ± 2.93	60.50 ± 13.87	88.90 ± 0.94	85.05 ± 1.75	61.11 ± 12.50
RUGS (Ours)	86.64 ± 1.69	84.10 ± 2.14	58.42 ± 13.47	12.42 ± 1.13	12.10 ± 2.63	13.84 ± 5.91	85.20 ± 2.74	82.46 ± 2.15	60.52 ± 13.04	89.76 ± 1.88	88.37 ± 1.91	61.44 ± 10.88

**Table 4:** Quantitative comparison of different methods on the BraTS-MEN 2023 dataset. Results are presented as mean ± standard deviation.

Method	DSC (%)			HD95 (mm)			Sensitivity (%)			Precision (%)		
	WT	TC	ET	WT	TC	ET	WT	TC	ET	WT	TC	ET
URPC [47]	74.23 ± 5.04	86.21 ± 6.33	76.78 ± 4.02	39.60 ± 8.85	18.65 ± 14.19	36.38 ± 8.16	72.22 ± 5.01	84.61 ± 7.06	76.60 ± 5.09	75.98 ± 4.32	92.89 ± 3.21	79.99 ± 2.92
ABD [48]	73.97 ± 4.63	85.94 ± 6.18	77.31 ± 3.87	38.15 ± 8.42	19.03 ± 13.84	34.52 ± 7.91	73.52 ± 4.81	84.73 ± 6.48	75.15 ± 4.92	76.53 ± 4.11	93.41 ± 2.95	79.68 ± 4.17
DPL [21]	62.40 ± 10.63	85.59 ± 7.14	66.68 ± 8.40	51.14 ± 9.96	20.08 ± 12.67	30.64 ± 10.45	65.61 ± 10.01	82.64 ± 8.02	64.17 ± 9.62	64.08 ± 7.56	90.89 ± 1.34	76.56 ± 2.72
UPL [22]	63.15 ± 9.85	80.25 ± 8.35	68.42 ± 7.91	48.95 ± 10.43	25.08 ± 11.20	31.15 ± 9.84	66.85 ± 9.32	81.10 ± 8.26	64.95 ± 9.28	63.50 ± 8.14	89.25 ± 2.15	77.40 ± 3.96
Source only	57.84 ± 38.86	24.99 ± 40.41	60.55 ± 39.56	37.31 ± 39.03	70.73 ± 44.02	32.87 ± 38.08	59.79 ± 39.98	23.66 ± 39.11	55.96 ± 39.26	62.63 ± 38.12	28.96 ± 44.51	76.27 ± 39.55
Target only	65.93 ± 3.18	78.92 ± 6.84	69.42 ± 3.25	58.45 ± 13.09	40.18 ± 23.50	53.25 ± 16.23	67.25 ± 6.50	77.79 ± 4.30	69.36 ± 5.36	69.54 ± 4.08	85.21 ± 10.79	73.40 ± 5.07
Fine-tune	78.24 ± 3.87	89.54 ± 5.91	80.82 ± 2.70	25.76 ± 7.86	13.44 ± 10.14	21.54 ± 6.25	78.02 ± 3.10	87.28 ± 6.90	79.24 ± 2.68	81.75 ± 5.23	94.82 ± 1.73	84.98 ± 3.28
Random	73.38 ± 5.17	86.99 ± 6.13	75.56 ± 4.14	45.17 ± 15.38	20.37 ± 16.65	43.37 ± 15.43	76.31 ± 2.28	85.44 ± 6.50	76.49 ± 2.79	74.27 ± 7.50	92.61 ± 4.30	77.54 ± 5.65
LC [46]	73.88 ± 4.69	87.20 ± 5.47	77.85 ± 3.25	44.89 ± 12.84	20.79 ± 25.44	30.77 ± 9.19	73.86 ± 2.60	84.17 ± 6.45	75.22 ± 3.54	78.51 ± 8.37	93.76 ± 1.58	84.46 ± 5.06
Core-set [31]	73.31 ± 4.64	87.82 ± 6.67	76.25 ± 3.83	46.15 ± 10.82	21.84 ± 18.30	39.64 ± 8.80	74.05 ± 4.49	85.37 ± 6.98	75.32 ± 4.64	75.28 ± 5.65	94.26 ± 3.21	79.12 ± 3.94
STDR [25]	73.35 ± 5.00	88.43 ± 6.02	76.79 ± 5.63	45.95 ± 2.66	24.08 ± 16.77	43.25 ± 7.59	75.74 ± 5.73	86.10 ± 7.12	77.05 ± 6.46	73.92 ± 5.15	94.25 ± 1.84	78.43 ± 5.39
UGTST [24]	73.52 ± 5.97	87.35 ± 6.09	76.32 ± 5.39	36.01 ± 16.80	22.46 ± 27.30	31.88 ± 11.83	71.96 ± 4.76	84.72 ± 6.72	73.58 ± 5.14	79.15 ± 7.07	93.82 ± 3.12	82.88 ± 5.03
RUGS (Ours)	76.23 ± 5.52	88.57 ± 5.42	78.87 ± 5.31	29.80 ± 9.10	18.40 ± 12.69	27.45 ± 10.90	74.87 ± 6.45	84.99 ± 6.58	76.21 ± 6.13	81.55 ± 6.01	96.05 ± 0.83	84.59 ± 5.42

**Table 5:** Domain adaptation performance of different models on the target domain (BraTS-SSA 2023) using the RUGS strategy. Results are presented as mean ± standard deviation.

Method	DSC (%)			HD95 (mm)			Sensitivity (%)			Precision (%)		
	WT	TC	ET	WT	TC	ET	WT	TC	ET	WT	TC	ET
UNet [12]	87.39 ± 3.78	85.04 ± 3.94	77.18 ± 4.03	10.54 ± 1.84	11.03 ± 1.88	11.69 ± 3.20	86.67 ± 5.11	84.59 ± 3.74	78.83 ± 4.19	89.17 ± 3.37	88.01 ± 3.65	78.83 ± 4.19
UNETR [44]	85.96 ± 3.34	79.97 ± 5.42	76.48 ± 4.95	20.61 ± 8.71	20.21 ± 7.74	18.87 ± 5.45	86.38 ± 3.89	80.39 ± 5.61	77.58 ± 4.07	86.84 ± 5.08	83.74 ± 5.86	78.40 ± 5.97
SwinUNETR-V2 [45]	88.15 ± 3.95	84.63 ± 5.24	78.44 ± 4.10	18.02 ± 7.00	13.87 ± 8.41	15.19 ± 8.72	88.27 ± 5.11	84.66 ± 4.04	80.52 ± 2.30	88.67 ± 3.27	87.23 ± 4.64	79.55 ± 4.57
TransAttUnet [9]	87.83 ± 2.71	84.23 ± 5.47	77.83 ± 3.44	12.03 ± 3.88	10.72 ± 3.71	11.25 ± 3.34	87.32 ± 5.86	82.88 ± 6.48	78.41 ± 3.04	89.23 ± 3.38	88.73 ± 3.49	81.64 ± 4.16
VM-UNet [11]	87.58 ± 3.72	82.30 ± 5.78	75.81 ± 2.88	11.80 ± 1.79	10.12 ± 3.49	11.13 ± 2.89	86.36 ± 4.12	82.41 ± 6.27	76.58 ± 3.25	89.74 ± 3.94	85.77 ± 5.24	77.62 ± 4.77
Swin-UMamba [13]	89.00 ± 2.97	85.95 ± 4.20	79.89 ± 4.62	10.56 ± 2.47	11.22 ± 3.01	11.15 ± 3.83	87.89 ± 4.07	84.88 ± 5.01	80.99 ± 3.55	90.91 ± 2.45	88.58 ± 3.29	82.30 ± 3.73
SFADA-Net (Ours)	90.21 ± 1.96	86.91 ± 4.72	82.16 ± 3.24	10.42 ± 2.66	8.91 ± 3.24	9.85 ± 3.86	89.17 ± 3.57	87.77 ± 4.89	82.40 ± 5.28	92.41 ± 1.73	89.48 ± 4.61	83.03 ± 3.48

**Table 6:** Ablation results for module effectiveness on the BraTS-2021 dataset. Results are presented as mean ± standard deviation.

Modules	DSC (%)			HD95 (mm)			Sensitivity (%)			Precision (%)		
	WT	TC	ET	WT	TC	ET	WT	TC	ET	WT	TC	ET
B	90.34 ± 8.12	88.49 ± 16.37	86.95 ± 13.48	8.06 ± 13.91	5.58 ± 9.37	3.52 ± 7.52	90.14 ± 11.81	90.09 ± 16.59	87.08 ± 15.19	92.10 ± 7.05	90.21 ± 14.86	88.49 ± 13.72
B + Mamba	90.72 ± 7.93	88.85 ± 16.20	87.61 ± 13.25	7.68 ± 12.67	5.05 ± 9.12	3.45 ± 7.40	89.95 ± 10.82	89.20 ± 17.13	87.35 ± 14.86	92.95 ± 6.82	91.45 ± 14.20	89.42 ± 12.58
B + SAPM	91.08 ± 7.64	89.04 ± 17.68	87.88 ± 13.14	7.35 ± 12.38	4.69 ± 9.17	3.38 ± 7.89	89.41 ± 11.03	88.33 ± 18.56	87.46 ± 15.03	93.96 ± 6.60	92.83 ± 14.04	90.30 ± 11.64
B + DPMKC	91.24 ± 7.54	89.32 ± 17.61	87.65 ± 14.25	7.11 ± 12.84	4.84 ± 10.59	3.70 ± 9.88	90.64 ± 10.98	89.81 ± 17.69	87.92 ± 15.65	92.92 ± 6.74	91.10 ± 16.24	89.08 ± 13.22
B + SAPM + DPMKC	92.23 ± 7.04	90.61 ± 15.46	88.73 ± 13.11	5.73 ± 9.81	3.96 ± 6.93	2.95 ± 6.12	91.42 ± 10.03	91.30 ± 15.20	89.56 ± 14.62	93.91 ± 6.38	92.23 ± 12.56	89.69 ± 11.73

B (baseline): the UNet-like segmentation architecture. Mamba: vanilla SSM-based module.

**Table 7:** Evaluation results of model complexity.

Model	Params (M)	FLOPs (G)	Inference Time (ms)
UNet [12]	31.04	41.95	4.82
UNETR [44]	87.70	20.27	5.88
SwinUNETR-V2 [45]	28.65	16.50	8.31
TransAttUnet [9]	22.65	68.02	7.74
VM-UNet [11]	34.62	5.80	14.18
Swin-UMamba [13]	55.06	33.70	11.24
B	23.77	30.07	5.62
B + Mamba	21.18	28.65	6.76
B + SAPM	23.01	29.74	8.72
B + DPMKC	24.98	30.84	6.58
B + SAPM + DPMKC (Ours)	24.22	30.5	10.17

B (baseline): the UNet-like segmentation architecture. Mamba: vanilla SSM-based module.

**Table 8:** Ablation results for the proposed RUGS strategy on the BraTS-MEN 2023 dataset. Results are presented as mean ± standard deviation.

Components	DSC (%)			HD95 (mm)			Sensitivity (%)			Precision (%)		
	WT	TC	ET	WT	TC	ET	WT	TC	ET	WT	TC	ET
B	62.15 ± 9.84	82.93 ± 7.26	63.59 ± 10.72	37.42 ± 12.95	20.57 ± 14.38	32.92 ± 13.84	59.35 ± 10.11	78.89 ± 8.43	58.83 ± 11.56	72.27 ± 8.37	94.11 ± 3.52	76.72 ± 9.18
B + Entropy	68.15 ± 8.29	84.65 ± 7.87	71.83 ± 8.00	46.33 ± 7.57	25.98 ± 18.71	45.46 ± 9.67	66.38 ± 8.57	84.20 ± 5.63	70.01 ± 9.78	73.50 ± 7.02	93.08 ± 4.07	76.23 ± 4.47
B + RUA	72.85 ± 6.14	85.90 ± 5.37	75.94 ± 6.82	36.28 ± 10.26	19.04 ± 11.47	39.28 ± 9.85	71.54 ± 6.88	85.21 ± 5.41	73.98 ± 7.14	76.77 ± 6.23	93.45 ± 2.91	80.38 ± 6.75
B + RUA + Core-set	76.48 ± 5.21	87.55 ± 5.39	77.39 ± 5.24	40.43 ± 13.40	20.34 ± 12.08	33.75 ± 14.20	77.44 ± 3.61	84.61 ± 5.64	75.92 ± 5.27	78.25 ± 8.06	94.32 ± 2.75	81.98 ± 6.11
B + RUA + UGCS	76.23 ± 5.52	88.57 ± 5.42	78.87 ± 5.31	29.80 ± 9.10	18.40 ± 12.69	27.45 ± 10.90	74.87 ± 6.45	84.99 ± 6.58	76.21 ± 6.13	81.55 ± 6.01	96.05 ± 0.83	84.59 ± 5.42

B (baseline): pseudo labels generated by the pre-trained model were used for adaptation. Entropy: samples with high prediction uncertainty were selected. Core-set: samples were selected based on diversity using a Core-set strategy.

## Data Availability

The data that support the findings of this study are publicly available from the BraTS challenge at https://www.synapse.org/ (accessed on 24 February 2025). Detailed information regarding data splits and preprocessing procedures is provided in the Experiments and Results section. The code used in this study is publicly available at: https://github.com/ZHW11/SFADA-for-brain-tumor-segmentation (accessed on 12 February 2026).
